# Efficacy of Supplemental Ultrasound-Guided Pericapsular Nerve Group (PENG) Block Combined with Lateral Femoral Cutaneous Nerve Block in Patients Receiving Local Infiltration Analgesia after Hip Fracture Surgery: A Prospective Randomized Controlled Trial

**DOI:** 10.3390/medicina60020315

**Published:** 2024-02-12

**Authors:** Seung-hee Yoo, Min-jin Lee, Min-hyouk Beak, Won-joong Kim

**Affiliations:** 1 Department of Anesthesiology and Pain Medicine, College of Medicine, Ewha Womans University Mokdong Hospital, Ewha Womans University, Seoul 07985, Republic of Korea; yoosh0710@naver.com (S.-h.Y.); minbeak@gmail.com (M.-h.B.); 2 Department of Anesthesiology and Pain Management, Yong-Chul Kim’s Pain Clinic, Seoul 03079, Republic of Korea; minjin137@naver.com

**Keywords:** hip fractures, interventional ultrasound, nerve blockades, opioid analgesics, postoperative pain, postoperative cognitive complications

## Abstract

*Background and Objectives*: Local infiltration analgesia (LIA) represents a potential approach to reducing pain in patients undergoing total hip arthroplasty (THA). The pericapsular nerve group (PENG) block also provides adequate analgesia for fractures and THA. As most hip surgeries use a lateral incision, affecting the cutaneous supply by branches of the lateral femoral cutaneous nerve (LFCN), the LFCN block can contribute to postoperative analgesia. However, no studies have investigated the effectiveness of supplemental PENG block combined with LFCN block in patients undergoing LIA after hip fracture surgery. Our study aimed to assess the effectiveness of PENG combined with LFCN block following hip fracture surgery in patients who underwent LIA. *Materials and Methods*: Forty-six patients were randomly assigned to LIA or PENG + LFCN + LIA groups. The primary outcome was the pain score at rest and during movement at 2, 6, 12, 24, and 48 h postoperatively. The total opioid dose for postoperative analgesia was also measured at the same time points. Secondary outcomes included postoperative cognitive function assessment. *Results*: The median pain scores at rest and during movement were lower in the PENG + LFCN + LIA group throughout the study periods compared to the LIA group, except at 2 h (at rest) and 48 h (during movement) after surgery. The total fentanyl dose was lower in the PENG + LFCN + LIA group at all time points after surgery when compared to the LIA group. Postoperative delirium incidence and the median abbreviated mental test scores were not significantly different between the two groups. *Conclusions*: The combination of PENG and LFCN blocks may contribute to enhanced recovery for patients undergoing LIA after hip fracture surgery. However, further well-controlled research is necessary to determine the effectiveness of supplemental PENG combined with LFCN block in addressing cognitive deficits in these patients.

## 1. Introduction

Hip fractures rank among the most common causes of hospitalization and disability in the geriatric population. This condition requires operation in approximately 98% of patients [1]. Effective postoperative pain management and early recovery are pivotal for favorable functional outcomes following hip surgery [2].

Local infiltration analgesia (LIA) entails injecting a substantial volume of a diluted, long-acting local anesthetic, with or without adjuvants, into the operative site [3,4]. A systematic review suggested that LIA presents a potential approach for reducing pain and analgesic consumption without increasing the risk of adverse events in patients undergoing hip arthroplasty [5]. Despite numerous studies emphasizing the enhanced efficacy of combining nerve blocks with LIA rather than employing LIA alone, the rationale for incorporating blocks into LIA has been questioned due to the impressive results demonstrated by LIA alone [6,7,8].

In 2018, Girón-Arango et al. introduced the pericapsular nerve group (PENG) block, using ultrasonography to target articular branches that reach the anterior hip capsule [9]. The PENG block was reported to offer adequate analgesia for fractures and dislocations of the hip joint, as well as total hip arthroplasty (THA) [9,10,11,12]. Additionally, as most hip surgeries use a lateral incision, areas not covered by the PENG block are affected regarding the cutaneous supply by branches of the lateral femoral cutaneous nerve (LFCN). Therefore, the LFCN block can contribute to postoperative analgesia [13].

A decline in postoperative cognitive function is a common challenge faced by hip fracture patients and has a multifactorial etiology [14]. Postoperative delirium (POD) incidence was reported in 16.9–28% of patients. Additionally, POD was associated with an increased 30-day mortality risk, prolonged hospital stays, difficulty in regaining daily function, and a higher risk of future cognitive dysfunction [15,16]. Furthermore, cognitive impairment is common in people with hip fractures. Up to 21% of individuals who require hip fracture repair present with dementia [17].

A robust association has been described between peripheral nerve block use and improvements in acute pain, delirium, and length of hospital stay following hip fracture surgery [18], particularly with supplemental peripheral nerve block [19]. However, to our knowledge, no studies have investigated the effectiveness of supplemental PENG combined with LFCN block in patients undergoing LIA after hip fracture surgery. This study hypothesizes that supplemental PENG and LFCN blocks will reduce postoperative pain, opioid consumption, postoperative delirium, and cognitive impairment after hip fracture surgery in patients undergoing LIA. Thus, our study aimed to investigate the effectiveness of PENG combined with LFCN block use following hip fracture surgery in patients undergoing LIA.

## 2. Materials and Methods

The Institutional Review Board of Ewha Womans University Hospital (EUMC 2021-07-002-006) approved this randomized controlled trial on 15 September 2021, which was subsequently registered with the Clinical Research Information Service (CRIS, http://cris.nih.go.kr, accessed on 21 October 2021, number: KCT0006682) on 21 October 2021. The first patient was enrolled on 24 November 2021. Each participant provided written informed consent before enrollment. The study adhered to the principles of the Declaration of Helsinki.

Sample size

A power analysis was conducted to estimate the sample size using G*Power version 3.1 (Heinrich Heine University, Düsseldorf, Germany). The pain score for patients undergoing THA at 2 h post-surgery, who received only LIA, was 2.53 ± 0.85 [20]. Assuming a 30% reduction in pain with supplemental PENG combined with the LFCN block compared to LIA alone, we calculated that 46 patients (23 per group, accounting for a dropout rate of 10%) were required, assuming a power of 0.80 and an α value of 0.05.

Patient recruitment

All patients aged ≥ 20 years diagnosed with unilateral hip fracture and scheduled for surgery were enrolled between November 2021 and May 2023. Inclusion criteria were a body mass index of 20–35 kg/m^2^ and an American Society of Anesthesiologists functional status of I–III. Exclusion criteria comprised patients with (1) other neuropathies in the hip joint; (2) chronic pain requiring opioid medication; (3) alcohol addiction; (4) previously diagnosed dementia; (5) mental illness or confusion; (6) admission to an intensive care unit; (7) previous open hip surgery; (8) intolerance of general anesthesia; (9) inability to communicate verbally or unwillingness to give informed consent; (10) coagulopathy or a tendency for bleeding; and (11) known allergies to the drugs used in this study.

Randomization

Patients were randomly assigned to LIA or PENG + LFCN + LIA groups using a computer-generated random number table. A research assistant not involved in patient care prepared the randomization list and opaque envelopes. An anesthesiologist, blinded to the study, recorded the outcome measures throughout the hospitalization. 

Performance of PENG combined with LFCN block and LIA

All patients received nasal cannula oxygen (2 L/min) and were placed in the supine position after routine skin sterilization. The nerve blocks were administered by the same anesthesiologist (YSH) before the induction of anesthesia.

For the PENG block, a low-frequency curvilinear probe (3–5 MHz) for ultrasound (Sonosite, Bothell, Washington, DC, USA) was positioned in a transverse orientation, medial and caudal to the anterosuperior iliac spine. A 23-gauge 5-inch spinal needle was advanced using an in-plane technique. It was directed laterally and then medially until its tip was positioned on the periosteum, dorsal to the psoas tendon [9]. A local anesthetic (20 mL, 0.375% ropivacaine) was injected following negative aspiration.

After the PENG block, a high-frequency linear probe (6–12 MHz) was used to identify the femoral artery under the inguinal ligament. The sartorius muscle and the LFCN, covered by the fascia between the sartorius and tensor fascia lata, were visualized. A local anesthetic (5 mL, 0.375% ropivacaine) was injected after negative aspiration.

The assessment of sensory loss in the anterior, medial, and lateral thigh compartments was conducted 30 min after the block. A successful block performance was defined as including the loss of pain sensation, evaluated through needle pinprick, and diminished cold sensations, assessed with an alcohol swap, compared to the contralateral side. Cases with unsuccessful block performances were excluded from the statistical analysis.

An orthopedic surgeon administered LIA to all patients for intraoperative analgesia. Before suturing the wound, an analgesic drug (0.1875% ropivacaine, 1:200,000 epinephrine, 30 mg of ketorolac, 1 mg of morphine sulfate, a total 80 mL) was injected around the joint capsule and into multiple sites, such as the exposed gluteus muscles and abductors, the peri-rotor region, and the subcutaneous tissue below the incision.

Anesthesia and postoperative analgesia

Following the nerve block, patients underwent general anesthesia administered by an anesthesiologist blinded to the allocation result. The induction included 1–2 mg/kg of 1% propofol, 1 µg/kg of remifentanil, and 0.6 mg/kg of rocuronium for endotracheal intubation. Anesthesia was maintained with a 1–1.5 minimum alveolar concentration of sevoflurane using 50% oxygen in the air, and remifentanil infusion was administered as needed. The bispectral index (BIS module, GE Healthcare, Helsinki, Finland) was maintained between 40 and 60, and systolic blood pressure and heart rate fluctuations were kept within 20% of the preoperative levels. Surgical interventions were performed by a single orthopedic surgeon using lateral approaches.

All patients received 200 mg of intravenous sugamadex at the end of the surgery, and intravenous patient-controlled analgesia (IV-PCA) was initiated before transferring patients to the post-anesthesia care unit. The PCA device (Accumate 1100^®^, Woo Young Medical, Seoul, Republic of Korea) consisted of 100 mL of PCA, comprising 16 µg/kg of fentanyl and 0.3 mg of ramosetron, delivered at a background flow rate of 0.5 mL/h, with a demand bolus of 0.5 mL and a lockout period of 15 min.

Outcome measurement

The primary outcome was the pain score, which was evaluated using the visual analog scale (VAS) at rest and during movement (hip flexion at 15°) at 2, 6, 12, 24, and 48 h postoperatively. VAS scores ranged from 0 to 100, indicating a gradual increase in pain. The total fentanyl dose of IV-PCA used for postoperative analgesia was also measured at the same time points.

Secondary outcomes included postoperative cognitive function assessment, measured on postoperative day 2 to account for potential residual anesthetic effects [21]. Suspicious symptoms of postoperative delirium (POD) were evaluated using the confusion assessment method (CAM), including nine criteria, with four considered “cardinal”: acute onset and fluctuating course, inattention, disorganized thinking, and an altered level of consciousness [22,23]. Abbreviated mental test score (AMTS), using a 10-point score based on verbal responses to 10 questions, was calculated and validated to detect any cognitive impairment in the geriatric population [24].

Statistical analysis

The distributional normality of continuous variables was assessed using the Shapiro–Wilk test. Parametric data were analyzed using the independent *t*-test and paired *t*-test. Non-parametric data were analyzed using the Mann–Whitney U-test and the Wilcoxon signed-rank test. Descriptive variables were evaluated using the χ^2^ test. Continuous variables were presented as mean ± standard deviation, and ordinal data and non-parametric data were expressed as median value (interquartile range) or number. Statistical significance was defined by *p* values < 0.05. All statistical analyses were conducted using PASW Statistics for Windows, version 18.0 (SPSS Inc., Chicago, IL, USA).

## 3. Results

**Figure 1:**
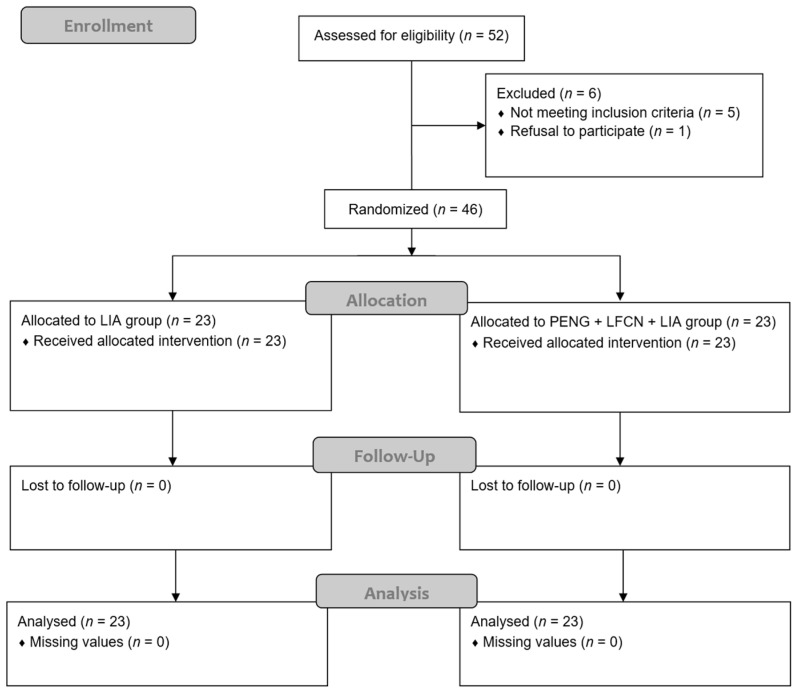
CONSORT flow diagram.

We recruited 52 patients that had been diagnosed with unilateral hip fractures and who were scheduled for surgery. Among them, five patients did not meet the inclusion criteria, and one patient refused to participate. Consequently, 46 patients were enrolled, with 23 assigned to the LIA group and 23 to the PENG + LFCN + LIA group, respectively (Figure 1). There were no patients lost to follow-up and no missing data at 48 h after treatment (Figure 1). Moreover, there were no significant differences in demographic data between the groups (Table 1).

Primary outcomes

**Figure 2:**
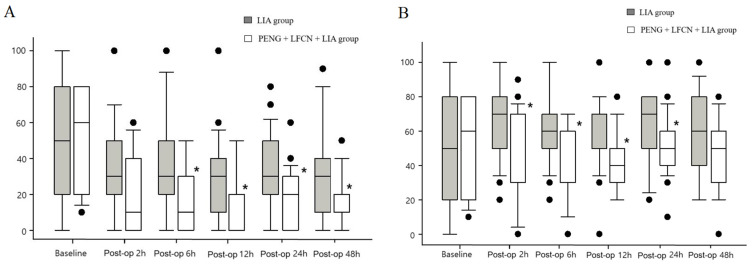
Postoperative pain scores (**A**) at rest; (**B**) during movement. * indicates a significant difference from LIA group (*p* < 0.05).

The median pain scores at rest and during movement were consistently lower in the PENG + LFCN + LIA group throughout the study compared to the LIA group, except at 2 h (at rest) and 48 h (during movement) after surgery (Figure 2). The total fentanyl dose administered via IV-PCA was lower in the PENG + LFCN + LIA group at all time points after surgery when compared to the LIA group (Table 2).

Secondary outcomes

POD occurred in one patient in the LIA group and four patients in the PENG + LFCN + LIA group without a statistically significant difference between the groups. Additionally, the median AMTS between the two groups was not significantly different (Table 3). None of the patients reported complications associated with the blockade.

## 4. Discussion

Patients who received supplemental ultrasound-guided PENG combined with LFCN block reported significantly reduced pain and opioid consumption at rest and during movement after hip fracture surgery compared to those receiving LIA alone.

The results regarding the clinical effectiveness of LIA alone and LIA combined with supplemental blocks varied between studies on total knee arthroplasty (TKA) and THA. For instance, the combination of LIA and adductor canal block (ACB) showed no significant differences in pain scores at rest, during movement, or in opioid usage during TKA [6]. Similarly, adding a saphenous nerve block to supplement LIA after TKA did not result in significant differences in pain scores on postoperative days 1 and 2 [8]. However, in the context of THA, patients who received a PENG block combined with LIA consumed significantly less morphine during the intraoperative and postoperative 24 h and had lower pain scores at rest and during motion within 24 h compared to LIA alone [25]. Additionally, ultrasound-guided anterior iliopsoas muscle space blocks combined with LIA provided better postoperative pain relief, decreased opioid consumption, and enhanced recovery after THA [26]. A systematic review of LIA in TKA and THA suggested that LIA might have limited additional analgesic efficacy in THA when combined with a multimodal analgesic regimen [27], thereby aligning with our results in patients undergoing hip fracture surgery.

The most popular techniques for postoperative analgesia in hip surgery comprise lumbar plexus blocks or psoas compartment blocks, fascia iliaca compartment blocks (FICBs), the “3 in 1” block, and distal nerve blocks, including the femoral nerve block (FNB) [28]. The PENG block, introduced by Girón-Arango et al. [9], effectively targets the articular branches of the femoral, obturator, and accessory obturator nerves supplying the anterior hip capsule. However, the articular branches of the femoral and accessory obturator nerves become consistently blocked, while the branches of the obturator nerve are not blocked consistently and are volume-dependent [29]. Histologically, the anterior hip capsule consists predominantly of nociceptive fibers, while the posterior capsule primarily comprises mechanoreceptors and lacks sensory fibers [30]. Therefore, the anterior capsule is highly innervated, emphasizing the importance of targeting these nerves for hip analgesia.

A scoping review [31] demonstrated that the potential advantages of the PENG block over traditional forms of regional analgesia for hip pain, such as the FNB, include broader and more complete coverage of sensory nerves innervating the hip. This broader coverage might lead to more effective regional analgesia and reduced postoperative pain [31,32]. In turn, this improvement might enhance patient satisfaction, decrease postoperative opioid consumption, and reduce opioid-related adverse events, as well as the likelihood of long-term opioid dependency [33]. Although this review identified heterogeneity in the PENG block regarding indications, combinations with other nerve blocks, different local anesthesia solutions used, differences in follow-up, and reporting of outcomes, a common practice involved using a high-volume, low-concentration dose (approximately 20–30 mL of 0.25% bupivacaine) [31].

In 1989, Dalens and colleagues first described the FICB, which simultaneously blocks the femoral nerve, obturator nerve, and LFCN of the thigh [34]. Recently, several systematic reviews and meta-analyses comparing PENG block and FICB for hip surgery have revealed somewhat inconsistent results. Andra et al. [35] suggested that the PENG block reduces opioid consumption during the initial postoperative 24 h and decreases the pain score at rest at 12 h postoperatively compared to FICB. Another analysis [36] showed no difference in pain scores at 6, 12, and 24 h between PENG and FICB, but the mean opioid consumption in morphine equivalents was significantly lower with PENG compared to FICB. Prakash et al. [37] observed no difference between the PENG block and FICB at 24 h for pain at rest and movement, while the PENG block showed improved analgesia within 30 min at rest and during movement, along with reduced postoperative opioid consumption within 24 h. Randomized controlled trials (RCTs) showed that the PENG block did not exhibit clinically significant differences in postoperative pain scores or cumulative opioid consumption compared to suprainguinal FICB [38,39].

The original objective of the PENG block was to develop a motor-sparing nerve block that could cover only the sensory afferent nerve fibers of the femoral, obturator, and accessory obturator nerves [28]. Giron et al. [9] demonstrated the better preservation of quadriceps muscle power in the postoperative period using the PENG block compared with FNB, which can be explained by the observation that the PENG block does not block the femoral nerve motor branches that innervate the quadriceps muscles. This finding is similar to the results of Short et al. [40]. Ghodki et al. [41] showed that normal quadriceps motor activity was found in only 13% of patients at 12 h postoperatively in patients receiving FNB. An RCT showed that the PENG block resulted in a lower incidence of quadriceps motor block and provided better preservation of hip adduction, as well as decreased sensory blocking of the anterior, lateral, and medial thigh, compared to suprainguinal FICB [38]. A meta-analysis [42] also highlighted the significantly decreased motor block of quadriceps muscle using the PENG block compared to other blocks.

Moreover, the easily identifiable sonographic landmarks of the anteroinferior iliac spine, the iliopubic eminence, and the psoas tendon make the technical performance of the PENG block comparable with other nerve blocks [9,43]. Indeed, the current literature supports the safety of the PENG block without reporting serious adverse events, such as permanent nerve injury, significant vascular damage, or local anesthetic systemic toxicity [31].

A narrative review [28] demonstrated that combining the PENG block with other blocks enhances operative analgesia. Pain after hip surgery arises from both the hip joint and soft tissues being disrupted during the surgical approach [19]. An RCT [13] and two case reports [44,45] indicated that blocking the LFCN might offer an additional advantage to the PENG block regarding the quality and duration of analgesia, especially since most hip fracture surgeries require a lateral incision. Consistent with existing studies, our results demonstrated that combining the PENG block with the LFCN block resulted in lower pain scores at rest and during movement and reduced opioid consumption after hip fracture surgery compared to LIA alone.

The most frequently utilized treatment for orthopedic pain was opioids. However, the use of opioids in older individuals was associated with numerous complications, including hypotension, prolonged hospitalization, respiratory depression, or postdischarge adverse effects such as dependence or addiction [46]. Specifically, high doses of opioids might contribute to an increased risk of confusion and delirium after surgery [47]. Higher doses of PCA-administered opioids were linked to a higher incidence of POD in individuals undergoing THA [48]. Additionally, high opioid consumption was reported in older adults undergoing surgery who received postoperative PCA and subsequently became delirious [49]. However, Sieber et al. [50] found no association between the use of postoperative opioids and incident delirium in participants with or without dementia after hip fracture repair. Morrison et al. [51] suggested that undertreated pain significantly contributes to delirium development. They argued that opioids do not precipitate delirium in patients with acute pain. Additionally, avoiding or administering very low doses of opioids was associated with an increased risk of delirium. In our study, although the pain score and opioid use were high in the LIA group, there was no difference between the groups regarding the incidence of POD and AMTS, possibly because CAM and AMTS were insufficient to assess postoperative delirium and confusion. Furthermore, other risk factors that could decrease postoperative cognitive function might not have been well controlled.

Our study had several limitations. First, our patients were not blinded, and subjects in the control group might have accurately guessed their group allocation. Second, the follow-up was limited to the hospital stay, precluding the evaluation of long-term effects after discharge. Third, one anesthesiologist and one orthopedic surgeon performed all nerve blocks and hip surgeries, respectively; thus, results might depend on clinical skill and experience. Fourth, the perioperative multimodal analgesia regimens used in our center might differ from those in other centers, potentially influencing the study’s results. Fifth, the relatively small sample limited the identification of differences in secondary outcomes. Sixth, the heterogeneity of surgical interventions could have introduced bias into the results; however, no significant differences were observed between surgery types. Finally, sensory loss was not assessed using standardized methods, such as quantitative sensory testing, to confirm the successful performance of the nerve block.

## 5. Conclusions

Median pain scores at rest and during movement were lower throughout most study periods in patients who underwent supplemental ultrasound-guided PENG combined with LFCN block than in those who received only LIA. Additionally, the total fentanyl dose was lower in patients with supplemental PENG combined with LFCN block at all time points after surgery when compared to those who received only LIA. Therefore, the combination of PENG and LFCN block might contribute to enhanced recovery for patients undergoing LIA after hip fracture surgery. However, the difference between the groups regarding POD and AMTS was not statistically significant. Well-controlled further research is needed to identify the efficacy of supplemental PENG combined with LFCN block on cognitive deficits in patients undergoing LIA after hip fracture surgery.

## Figures and Tables

**Table 1:** Demographic data.

	LIA Group (*n* = 23)	PENG + LFCN + LIA Group (*n* = 23)	*p* Value
Age (years) *	70.87 ± 13.42	73.68 ± 12.99	0.385
Height (cm) *	159.40 ± 12.36	157.57 ± 8.55	0.668
Weight (kg) *	60.99 ± 11.46	56.18 ± 10.85	0.794
Sex (M/F) ^†^	7 (30)/16 (70)	6 (26)/17 (74)	0.743
Operation time (min) *	66.96 ± 36.11	62.04 ± 19.920	0.499
Anesthesia time (min) *	112.39 ± 35.67	114.77 ± 28.51	0.891
ASA (1/2/3) ^†^	2 (9)/9 (39)/12 (52)	1 (4)/9 (39)/13 (57)	0.830
Location (right/left) ^†^	8 (35)/15 (65)	10 (43)/13 (56)	0.546
Fracture site Femur neck/intertrochanter ^†^	18 (78)/5 (22)	15 (65)/8 (35)	0.326
Type of surgery THA/hemiarthroplasty/internal fixation ^†^	14 (60)/2 (9)/7 (31)	12 (52)/2 (9)/9 (39)	0.817
Estimated blood loss (mL) *	371.74 ± 178.26	374.09 ± 211.25	0.779
Intraoperative remifentanil (µg) *	256.52 ± 171.43	202.50 ± 121.08	0.225

* Values are expressed as the mean ± standard deviation, and ^†^ indicates the number (%) of patients. ASA: functional status according to the American Society of Anesthesiologists; THA: total hip arthroplasty.

**Table 2:** Postoperative total opioid use.

Total Fentanyl Dose (µg)	LIA Group (*n* = 23)	PENG + LFCN + LIA Group (*n* = 23)	*p* Value
Postoperative 2 h *	27.96 ± 8.80	22.11 ± 8.61	0.029
Postoperative 6 h ^†^	59.40 (30.30)	38.16 (21.00)	0.001
Postoperative 12 h ^†^	118.03 (49.74)	72.16 (35.88)	<0.001
Postoperative 24 h ^†^	204.00 (74.40)	138.16 (54.15)	<0.001
Postoperative 48h ^†^	382.80 (170.36)	273.20 (95.90)	0.003

* Values are expressed as the mean ± standard deviation, and ^†^ indicates the median (interquartile range).

**Table 3:** Postoperative cognitive functions.

	LIA Group (*n* = 23)	PENG + LFCN + LIA Group (*n* = 23)	*p* Value
POD incidence *	1 (4)	4 (17)	0.155
AMTS ^†^	7.00 (2.00)	8.00 (2.50)	0.234

* Values are expressed as the number (%) of patients, and ^†^ indicates the median (interquartile range). AMTS: abbreviated mental test scores; POD: postoperative delirium.

## Data Availability

The datasets produced and/or examined in the course of this study can be obtained upon reasonable request from the corresponding author.
